# The Rubber Question

**Published:** 1881-06

**Authors:** R. Finley Hunt


### ARTICLE IV.

### *The Rubber Question.*

BY R. FINLEY HUNT, D. D. S.

I avail myself with pleasure of your proposition that I should write for your new journal an article on the Cummings patent. This enables me to answer to the profession generally many inquiries that I have received from individual correspondents, and while those to whom I have replied are in possession of many points in this paper, I am satisfied there is a large number to whom they will be new and interesting. Under the circumstances this ought to be an old story, familiar in all its details to every dentist in the country; but it is not so. Not one dentist in a hundred can give an intelligent statement of the matter as it has been and as it stands now. A very large majority of them only know that they have been called upon to pay fifty, seventy-five and some two or three hundred dollars per annum for the use of hard rubber in making dental plates in the practice of their profession. And while they felt that the claim for the payment of these sums was unjust, they have, nevertheless, paid them, not, however, without protests and contests, but their efforts have been ineffectual; and why? *Because there has been no proper union and concert of action on the part of members of the profession, and their defence has been entrusted too much to others.* It is true that there have been occasional spasmodic efforts at organized resistance, but these have been spasmodic in that they were only made to meet an immediate and unforeseen attack. What organization there was, was confined to the locality where the attack was made, and too much reliance was placed in lawyers (with all due deference to that profession) and others whose interests were not identified with those of dentists, the ones having most at stake.

Look at the result of this want of proper organization, union and concert of action. *The dental profession has paid to the Goodyear Dental Vulcanite Company upwards of three million dollars!* One-twentieth part of this enormous sum properly and intelligently expended would have freed the dentists from the payment of any royalty or license for the use of hard rubber for dental plates from and after May 5, 1872, the date of the expiration of the Goodyear patent. This expression of opinion is based upon my thorough acquaintance with, and study of this subject from 1866 to the present time. It is true that a large amount of money has been expended and a great deal of work done in contesting the validity of the Cummings patent, but it was the contest of an unorganized multitude, comparatively, against a thoroughly and compactly organized few, and, as remarked before, there were no preparations for resistance before the attack had been made Consequently, every prosecution against dentists for infringment of the Cummings patent has been what would be called in military parlance a "surprise." It is not, therefore, to be wondered at that the dentists have suffered so many defeats, particularly in view of the fact that in all litigation, as a general rule, the client must furnish the facts of a case while the counsel applies the law to it. Here the defendants were not cognizant of the facts important to their defence, and in their absence the courts were bound to decide, *on the evidence before them*, in favor of the Goodyear Dental Vulcanite Company. The real facts were not brought to light until after several decisions made on this imperfect defence led the courts to consider the matter a *res adjudicata*, and to view it as against public policy to disturb the decrees deliberately made.

But all this now belongs to the dead past, and in this progressive age we cannot afford to spend time in *post mortems.* I have written all this preparatory to the important questions that follow. Is the profession prepared to offer successful resistance to aggressions of this or other monopolies? Is it not a dictate of common sense, as well as a duty, to profit by the past and guard against the extortion of other millions in the future from the pockets of its members? Can this be done, and if so, how?

It may be said that the Cummings patent will expire June 9, 1881, and we will then be free from its extortions, but dentists have been warned that an attempt would be made to have it extended by special act of Congress. Are we properly prepared to meet and defeat such an attempt? The Goodyear Dental Vulcanite Company are, moreover, equitable owners of the patent granted to Robert Haering, January 19, 1869, for a term of 17 years, extending to January 19, 1886, for "artificial teeth having gum bases, to which the teeth are secured as described; that is, by setting the teeth in prepared gum on a mould, imbedding the whole in plaster and hardening the gum by the application of heat." This is substantially the same as the Cummings patent, and the process described in the specifications is the same as that used by every dentist who makes a set of teeth on hard rubber. With the possession of this patent and the chances of an extension of the Cummings patent, (which they can obtain if not properly opposed,) is it reasonable to suppose that the G. D. V. Co. will relinquish without an effort an income of upward of three hundred thousand dollars ($300,000) per annum? Is it not rather probable that under the same name or in some other guise they will endeavor to continue their extortions from a class of men whom they have found hitherto so easy a prey?

For self-protection in this and all other matters I propose that the profession organize in each one of the States by Congressional Districts, with a good State Committee. Then let there be a General Committee, to whom may be entrusted the management and control of the defence of the profession against all aggressions. The fund for this purpose to be raised by an annual contribution of fifty cents or one dollar by each and every dentist, and placed in the hands of the General Committee. This is but the merest skeleton of the proposed organization. It would require much consultation and work to complete it, but it would pay, and pay well for all the labor bestowed upon it. If it were put in operation a contributing member of the organization who might be attacked need only send a proper notification to his State Committee who, with the General Committee, would take charge of his defence and leave him to attend to his regular business.

I hope that this matter will be discussed this season at all the meetings of State and other dental associations, and result in more closely uniting the profession of the country for concentrated action in this matter.—*Herald of Dentistry.*

